# Regulation of zebrafish sleep and arousal states: current and prospective approaches

**DOI:** 10.3389/fncir.2013.00058

**Published:** 2013-04-09

**Authors:** Cindy N. Chiu, David A. Prober

**Affiliations:** Division of Biology, California Institute of TechnologyPasadena, CA, USA

**Keywords:** zebrafish, sleep, arousal, hypocretin, orexin, neuromodulator

## Abstract

Every day, we shift among various states of sleep and arousal to meet the many demands of our bodies and environment. A central puzzle in neurobiology is how the brain controls these behavioral states, which are essential to an animal's well-being and survival. Mammalian models have predominated sleep and arousal research, although in the past decade, invertebrate models have made significant contributions to our understanding of the genetic underpinnings of behavioral states. More recently, the zebrafish has emerged as a promising model system for sleep and arousal research. Here we review experimental evidence that the zebrafish, a diurnal vertebrate, exhibits fundamental behavioral and neurochemical characteristics of mammalian sleep and arousal. We also propose how specific advantages of the zebrafish can be harnessed to advance the field. These include tractable genetics to identify and manipulate molecular and cellular regulators of behavioral states, optical transparency to facilitate *in vivo* observation of neural structure and function, and amenability to high-throughput drug screens to discover novel therapies for neurological disorders.

## Introduction

Animals engage in diverse activities that require adaptive changes in behavior. A fundamental goal in neuroscience is to understand how the brain enables animals to make dynamic changes in behavioral state in response to changing internal or environmental demands. A particularly striking example of such a change in behavioral state is the switch between sleep and wakefulness. Once awake, animals must further modulate arousal levels—for example, transitioning between inattentive and attentive states—as required for the task at hand. Underscoring the significance of these behavioral states, sleep and arousal states are conserved across the animal kingdom, from worms and flies to fish and humans (Allada and Siegel, 2008; Cirelli and Tononi, 2008). Despite the severe consequences and prevalence of sleep and arousal disorders (Mahowald and Schenck, 2005), the mechanisms that regulate behavioral states and transitions between states remain mysterious.

Theories to account for the regulation of sleep and arousal states span the hierarchy of biological organization, from organismal physiology, behavior, and cognition to neurons and neural ensembles, and more recently to genetic and molecular mechanisms (Hobson and Pace-Schott, 2002; Pace-Schott and Hobson, 2002). The zebrafish, which offers experimental advantages at many levels, is well-suited to contribute to our understanding of these states. These advantages include a simplified yet conserved vertebrate brain, facile genetics, an increasingly well-characterized behavioral repertoire, amenability to pharmacological and high-throughput assays, and optical transparency for *in vivo* visualization of the brain (Lieschke and Currie, 2007). The zebrafish is also gaining traction as a useful system for circuit neuroscience (Friedrich et al., 2010; McLean and Fetcho, 2011; Portugues et al., 2013).

In this review we will survey key concepts and open questions in the field of sleep and arousal regulation and then examine current approaches to identifying these behavioral states in zebrafish. To exemplify these concepts and the issues that arise when using zebrafish to study neuromodulation of sleep and arousal, we will focus our discussion on studies that explore the role of hypocretin, an important mammalian neuromodulator of sleep and arousal, in regulating zebrafish behavioral state. Finally, we will highlight recent innovations in the zebrafish toolkit that have the potential to open new avenues of discovery in sleep and arousal research.

## Regulation of sleep and arousal: key concepts and problems

In the early twentieth century, the neurologist Constantin von Economo examined encephalitis patients suffering from profound sleep disorders. He discovered that excessive sleepiness was associated with a specific pattern of brain lesions located at the junction of the brainstem and forebrain, whereas insomnia was associated with lesions in a nearby, more anterior region (von Economo, 1930). Subsequently, Moruzzi, Magoun, and others found that sleep or arousal could be induced by lesion or electrical activation along a subcortical pathway ascending from the brainstem (Moruzzi and Magoun, 1949). These findings advanced the idea that sleep and arousal states are actively generated and maintained by the brain. The main subcortical regions identified by von Economo and others (brainstem, posterior hypothalamus, basal forebrain) are now known to contain distinct aminergic and peptidergic cell populations (Saper et al., 2005). These systems promote arousal via ascending projections that increase forebrain excitation as well as descending brainstem and spinal cord projections that increase muscle tone and sensorimotor function (Jones, 2003).

**Figure 1:**
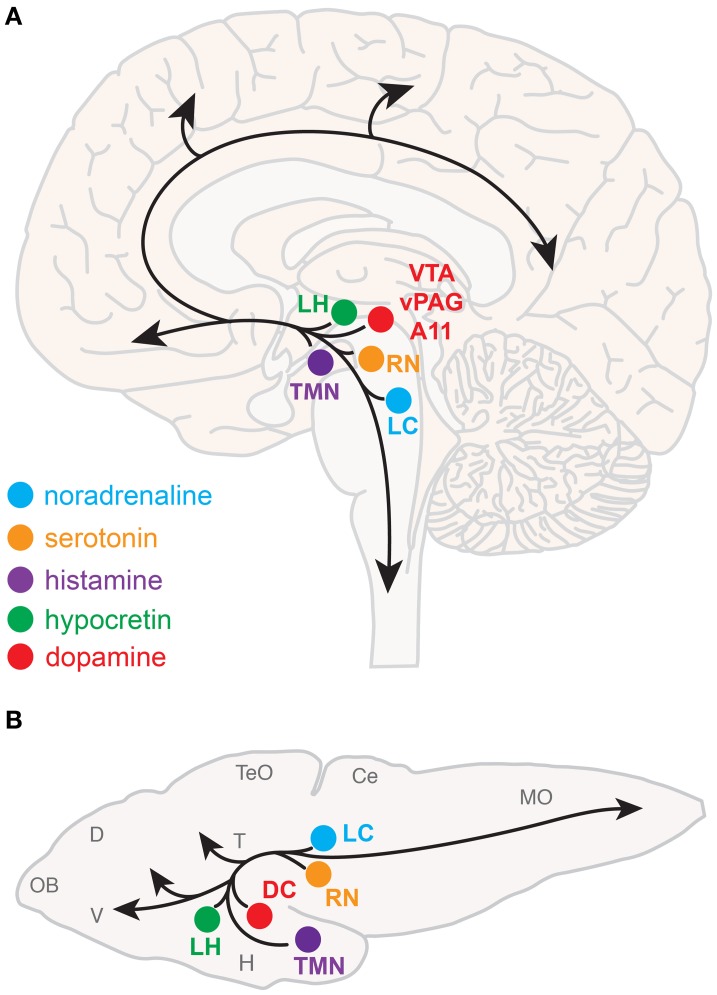
**Neuromodulatory systems that promote arousal in vertebrates.** The approximate locations of key neuromodulatory regions are shown for human **(A)** and larval zebrafish **(B)** brains. Arrows indicate ascending projections that increase forebrain excitation and descending projections that increase muscle tone and sensorimotor function. Abbreviations of neuromodulatory regions: LC, locus coeruleus; RN, raphe nuclei; VTA, ventral tegmental area; vPAG, ventral periaqueductal gray; A11, mammalian dopamine cell group A11; DC, dopaminergic diencephalic cluster; TMN, tuberomammillary nucleus; LH, lateral hypothalamus. Abbreviations of larval zebrafish brain anatomy: OB, olfactory bulb; D, dorsal telencephalon; V, ventral telencephalon; TeO, optic tectum; H, hypothalamus; T, thalamus; Ce, cerebellum; MO, medulla oblongata. Note human and larval zebrafish brains are not depicted to scale.

Neuromodulatory systems that promote arousal include (refer to Figure 1A):

- Noradrenergic neurons of the locus coeruleus, located in the pontine brainstem;
- Serotonergic neurons of the raphe nuclei, located in the midbrain;
- Dopaminergic neurons, particularly those of the ventral periaqueductal gray (vPAG) and ventral tegmental area (VTA) of the midbrain, and also the A11 dopaminergic cell cluster, located in the hypothalamus;
- Histaminergic neurons of the tuberomammillary nucleus, located in the posterior hypothalamus;
- Hypocretin (Hcrt) neurons located in the lateral hypothalamus;
- Cholinergic neurons located in the basal forebrain and also in the pedunculopontine and laterodorsal tegmental nuclei, located in the pontine brainstem.

In contrast to the many known arousal-promoting systems, attempts to identify distinct sleep-promoting cell populations have been less fruitful. One exception is the ventrolateral preoptic area (VLPO), which was identified as a cluster of cells in the basal forebrain which provides inputs to systems in the hypothalamus and brainstem that promote arousal (Saper et al., 2005). These neurons are likely the site of lesion in von Economo's insomnia patients, and subsequent work in animal models showed that VLPO lesions reduce sleep by more than half (Lu et al., 2000). VLPO neurons contain the inhibitory transmitters GABA and galanin, and they are likely to promote sleep by inhibiting arousal systems. The VLPO, in turn, is directly and indirectly inhibited by arousal systems (Saper et al., 2005).

A crucial question is how these neural populations operate together to regulate distinct states and the transitions between them. The bistable flip-flop switch, adopted from electronics theory, is one appealing circuit-level model that can explain the rapid transition between distinct behavioral states such as sleep and waking (Saper et al., 2001). The flip-flop circuit derives its features from two reciprocally inhibitory components; this could be implemented in the brain by mutual inhibition between the VLPO and arousal systems. Because the flip-flop switch is inherently unstable, the circuit model might be supplemented by additional elements (i.e., neuromodulatory systems) that serve to stabilize and sustain a wake or sleep state. For example, in mammals, the neuromodulator Hcrt might serve to stabilize sleep-wake states by promoting arousal. Indeed, loss of Hcrt signaling is a hallmark of narcolepsy, a disorder characterized by fragmented sleep-wake states.

A more phenomenological but influential model proposes that sleep is regulated by two main drives: homeostatic drive (also known as “Process S”) that is regulated by internal cues and circadian drive (“Process C”) that is regulated by environmental cues (Borbély et al., 1989). Genetic approaches have made remarkable contributions toward a molecular-level understanding of Process C. The core mechanism of the circadian clock is conserved across species and consists of a network of positive and negative molecular feedback loops that can cell-autonomously maintain a 24-h periodic rhythm (Zhang and Kay, 2010; Mohawk et al., 2012). In many animals, neurons of the hypothalamic suprachiasmatic nucleus (SCN) function as a “master clock” that orchestrates organismal circadian physiology and behavior. Despite our mechanistic understanding of the circadian clock, it remains unclear how the circadian system regulates behaviors associated with sleep and wakefulness, although secreted peptides such as prokineticin 2 and transforming growth factor alpha/epidermal growth factor appear to play key roles (Kramer et al., 2001; Cheng et al., 2002; Foltenyi et al., 2007; Van Buskirk and Sternberg, 2007; Gilbert and Davis, 2009). Our understanding of mechanisms that underlie Process S are more limited. One hypothesis is that adenosine, which accumulates as ATP energy stores are depleted during wakefulness, might serve as a signal for sleep need (Porkka-Heiskanen and Kalinchuk, 2011). Indeed, extracellular adenosine levels rise in specific regions of the mammalian brain during prolonged wakefulness and decline during sleep (Porkka-Heiskanen et al., 1997), and pharmacological activation of adenosine signaling promotes sleep (Benington et al., 1995; Hendricks et al., 2000; Thakkar et al., 2001; Rihel and Schier, 2012) and activates the VLPO (Scammell et al., 2001; Gallopin et al., 2005). However, the role of adenosine in sleep remains controversial because adenosine receptor mutants exhibit relatively normal sleep/wake behaviors (Stenberg et al., 2003; Urade et al., 2003; Huang et al., 2005; Bjorness et al., 2009; Wu et al., 2009).

A common theme that has emerged from studies of sleep and arousal regulatory mechanisms is that they play multiple roles in animal behavior and physiology. For example, many of the key players in sleep and circadian function are linked to metabolic regulation (Adamantidis and de Lecea, 2009; Bass, 2012). In fact, at the same time that Hcrt's link to narcolepsy was discovered (Chemelli et al., 1999; Lin et al., 1999), this peptide was also given the name orexin because intracerebroventricular injection of the peptide induced voracious feeding in rodents (Sakurai et al., 1998). Also, there are well-documented links between obesity and abnormal circadian behaviors, including voluntary behaviors such as shift-work (Antunes et al., 2010). This has led to the hypothesis that the circadian clock coordinates various physiological and behavioral functions in addition to sleep, such as liver function and feeding. Additionally, a number of sleep regulators have known interactions and/or overlap with regulators of immune function (Krueger, 2008) and learning and memory (Harris and Aston-Jones, 2006). Similarly, memory, attention, anxiety, and depression are among the many behavioral processes linked to arousal regulation and dysregulation (Johnson et al., 2012), and several neurological disorders, including autism and schizophrenia, are associated with sleep and arousal defects (Glickman, 2010; Pritchett et al., 2012).

Understanding how sleep and arousal are regulated might lead to new treatments for neurological diseases as well as explain normal individual variations in sleep and arousal. Going forward, a few of the many outstanding questions regarding how behavioral states are regulated include:

- What are the undiscovered genetic and neural substrates of sleep and arousal states?
- Do conserved or diverse neural and genetic mechanisms regulate sleep and arousal throughout the animal kingdom?
- What are the downstream effectors of “Process S” and “Process C”? For example, what are the neural and genetic pathways that link circadian input signals to a circadian behavioral output?
- How are circadian, homeostatic, and other behaviorally-relevant drives integrated at the circuit level?
- Are dynamic changes in neuromodulatory influences responsible for the transitions between different behavioral states?
- Which properties are unique and which are shared among neuromodulator systems that regulate sleep and arousal?
- Are there anatomical and functional subdivisions within each arousal-promoting neuromodulator system?
- Can we develop or discover effective remedies for sleep and arousal-related disorders? In particular, can we learn enough about mechanism to treat specific pathologies without grossly affecting other brain functions?

Despite their tremendous contributions to sleep and arousal research, prevalent animal model systems have limitations in addressing some of these questions. For example, drawbacks of rodent model systems include the relative complexity of their nervous systems, the difficulty of monitoring the activity of genetically identified neurons during behavior, and a nocturnal sleep/wake pattern, which differs from diurnal humans. Also, the long generation time, small litter size, and expense makes the rodent an unwieldy model for large-scale behavioral screening. On the other hand, the fruit fly and the worm are particularly amenable to genetic screens, but their nervous systems lack structures and some neuromodulators analogous to mammals. In this light, the zebrafish, a diurnal vertebrate with cutting-edge genetic and *in vivo* neuroimaging capabilities and a successful track-record in high-throughput behavioral screens, is an excellent system to complement the advances made using mammalian and invertebrate model systems.

## Analysis of zebrafish behavioral states

After only a few days of development, larval zebrafish begin to swim around in their environment, typically in brief, phasic locomotor episodes. High-speed infrared video capture combined with computational image analyses have been used to quantitatively describe specific locomotor behaviors in larval zebrafish. For example, by measuring values for indicator variables, such as those characterizing an animal's posture (tail bend location and amplitude, turning angle, yaw) and timing (tail-beat frequency, swimming speed), it is possible to objectively define and differentiate basic locomotor modules such as scoots, burst swims, routine-turns, and escape-turns (Budick and O'Malley, 2000).

Similarly, an animal's behavioral state can be defined as a *“recurring, temporally enduring constellation of values of a set of indicator variables of the organism”* (Steriade and McCarley, 2005). Sleep and waking states are typically defined in this manner. In humans and other mammals, these states can be distinguished by obvious differences in behavior, but they are more conveniently identified by objective electrophysiological measures that correlate with behavioral state, such as the electroencephalogram (EEG), which measures cerebral electrical activity (Berger, 1929). In fact, the EEG and similar measures of global brain activity reveal physiological subdivisions within sleep and waking, suggesting that they are not unitary states (Lin and Gervasoni, 2008). The use of physiological criteria is a practical and standardizable approach to defining behavioral states (Rechtschaffen and Kales, 1968; Datta and Hobson, 2000), although it is associated with the hazards of inferring cause from inappropriate or indirect measures (Hobson and Steriade, 1986). Indeed, when studying mammalian sleep early in ontogeny before adult EEG signatures are established, one must rely on mostly behavioral criteria (Blumberg et al., 2005). With this in mind, behavioral measures are more appropriate for describing behavioral states, whereas physiological measures of sleep and arousal, while experimentally convenient in some animals, should be carefully regarded as correlative.

**Figure 2:**
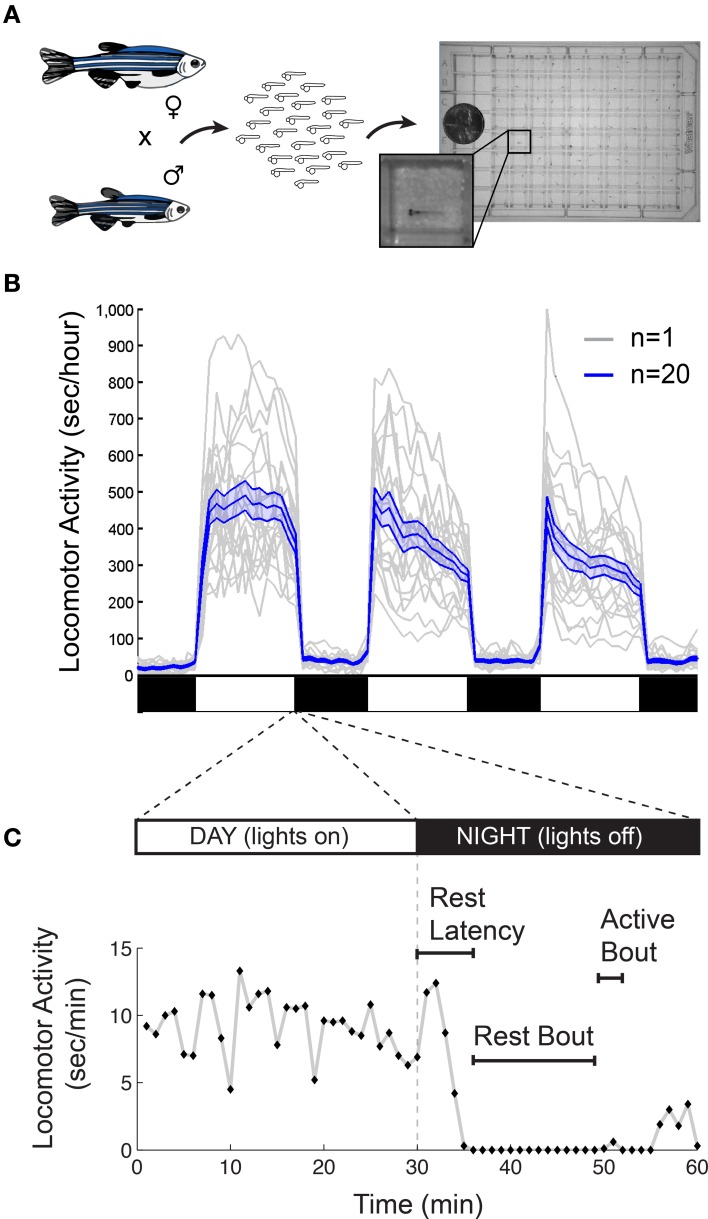
**Monitoring larval zebrafish sleep and wake behaviors. (A)** Zebrafish larval locomotor activity assay. Individual zebrafish larva are placed in each well of a 96-well-plate on the 5th day of development. The plate is placed in a temperature-controlled chamber that is illuminated by white lights during the day and is continuously illuminated by infrared lights. The larvae are monitored by an infrared camera and the locomotor activity of each larva is recorded by a computer. **(B)** Representative locomotor activity data for each of 20 individual wild-type larvae (gray traces) and their mean locomotor activity (blue trace, ±standard error of the mean) is shown. Black and white bars indicate day and night, respectively. Larvae are more active during the day than at night, although there is considerable variability among individuals. **(C)** An example of typical larval zebrafish behavior at the end of the day is shown. A rest bout is defined as a period of at least 1 min of inactivity, which is associated with an increase in arousal threshold (Prober et al., 2006). Rest latency indicates the time between lights off at night and initiation of the first rest bout. Figure modified from Prober et al. (2006).

There are also important methodological issues to address when measuring behavioral states in non-human animals including zebrafish. First, tracking of individual rather than groups of animals is ideal for resolving the temporal structure of sleep and arousal states. Second, the impact of genetic variations (Valatx et al., 1972) and prior experiences (Ganguly-Fitzgerald et al., 2006) on sleep and arousal must be carefully controlled for reproducible measurements. Fortunately, with care, these issues can be reasonably addressed using zebrafish (Figure 2A). Large clutches of embryos allow for experimental comparisons of siblings that are raised and tested together in identical conditions. Because they can survive on their yolk sac for the first week of development, the confounding effects of variable feeding behavior are avoided. Most importantly, the small size of larval zebrafish (~4 mm in length) allows for simultaneous behavioral tracking of individually-housed animals in a 96-well-plate.

### Zebrafish sleep states

In non-mammalian and non-avian animal model systems, sleep is defined according to several behavioral criteria (Campbell and Tobler, 1984): (1) quiescent state regulated by a circadian rhythm, (2) reduced sensory responsiveness, and (3) homeostatic regulation. Based on these criteria, behavioral sleep states have been demonstrated in flies and worms (Hendricks et al., 2000; Shaw et al., 2000; Van Buskirk and Sternberg, 2007; Raizen et al., 2008), and indeed, a number of studies have documented at least the first criterion for behavioral sleep in many fish species (Reebs, 1992). Recently, larval and adult zebrafish have been reported to exhibit all three behavioral criteria for sleep (Zhdanova et al., 2001; Prober et al., 2006; Yokogawa et al., 2007).

#### Criterion No. 1: quiescent state regulated by circadian rhythm

Starting around 4 days post fertilization (dpf), zebrafish raised on a 24-h alternating light: dark cycle (e.g., 14 h light:10 h dark) exhibit daily fluctuations in locomotor activity (Hurd and Cahill, 2002; Prober et al., 2006). Like humans, zebrafish are diurnal and thus exhibit peak activity during the light phase and increased quiescence during the dark phase (Figure 2B). Particularly at night, zebrafish spend bouts of several minutes or longer in a state of inactivity (Figure 2C).

As has been observed in many other animals, larval and adult zebrafish that have been entrained on a light: dark cycle maintain circadian oscillations in locomotor activity even after external circadian cues are removed (Hurd et al., 1998; Hurd and Cahill, 2002). The core molecular machinery of the mammalian circadian clock is well-conserved in zebrafish [reviewed in Vatine et al. (2011)], although zebrafish possess two paralogs of some mammalian genes (Postlethwait et al., 1998). A notable difference between zebrafish and mammals is that zebrafish peripheral circadian clocks are directly entrainable by light (Whitmore et al., 2000; Pando et al., 2001), a function that may have evolved in zebrafish due to their relative transparency. This innovation suggests that zebrafish may not require a “master clock” analogous to the mammalian SCN to orchestrate circadian rhythms throughout the body. We also note that some widely-used inbred strains of laboratory mice lack enzymes required to synthesize melatonin (Ebihara et al., 1986; Goto et al., 2007), a hormone produced in the pineal gland that is thought to play a key role in transmitting circadian cues in humans and zebrafish. Thus, the role of the pineal gland and melatonin might be underestimated in mammalian research using these laboratory mouse strains. This fact, together with the diurnal sleep/wake pattern of zebrafish, suggests that zebrafish have some important advantages over rodents for modeling the circadian regulation of human sleep.

#### Criterion No. 2: reduced sensory responsiveness

Sleeping animals exhibit reduced responsiveness to sensory stimuli, which distinguishes sleep from quiet wakefulness. During quiescent periods, larval zebrafish show reduced responsiveness to mechanical stimuli (Zhdanova et al., 2001) and delayed responses to sudden changes in light intensity (Prober et al., 2006), and quiescent adult zebrafish are less responsive to electrical stimuli (Yokogawa et al., 2007). Because larval zebrafish exhibit reduced responsiveness after at least 1 min of inactivity, sleep in larval zebrafish has been operationally defined as a quiescent bout lasting at least 1 min. A similar approach has been used to define sleep in adult zebrafish as a minimum 6-s inactive bout (Yokogawa et al., 2007). Presently, it is unclear whether differences between adult and larval zebrafish sleep reflect true differences in biological phenomena or merely differences in methodology across different studies; further experiments using standardized approaches across different zebrafish developmental stages should clarify this issue. Nonetheless, this approach to defining sleep states has been useful for identifying evolutionarily conserved sleep regulators in zebrafish (see next section). However, additional work may further refine the definition of zebrafish sleep by using detailed assays of physiology and arousal across various sensory modalities during quiescence. For example, one report indicates that quiescence during day and night are not equivalent based on the observation that nighttime quiescence is associated with reduced respiration and postural changes compared to daytime quiescence (Zhdanova, 2006).

#### Criterion No. 3: homeostatic regulation

A common approach to assaying homeostatic regulation of sleep is to test whether compensatory sleep occurs following a period of deprivation. Indeed, both larval and adult zebrafish exhibit this so-called “sleep rebound” behavior. In a study of larval zebrafish (Zhdanova et al., 2001), a vibration stimulus applied during the last 6 h of the night resulted in sleep rebound the following day. The reduced locomotor activity during sleep rebound was accompanied by a significantly decreased sensitivity to a mechanical stimulus as compared to siblings not subjected to sleep deprivation. In a study of adult zebrafish (Yokogawa et al., 2007), electroshock or light stimuli applied for 6 h at night reduced locomotor activity the following day, although arousal threshold was not assessed. Notably, sleep rebound in zebrafish has only been observed in dark testing conditions (Yokogawa et al., 2007).

Although these data are suggestive of rebound sleep, further advances in both technique and knowledge are needed to firmly establish homeostatic control of sleep in zebrafish. One important consideration in the design of sleep deprivation studies is the possibility of off-target effects of the deprivation protocol. For example, while light is a profoundly arousing stimulus for zebrafish, its utility as a specific sleep deprivation stimulus is limited because light also affects the circadian clock. Another confounding effect of sleep deprivation that can vary with different deprivation protocols is stimulus-induced stress, which may be caused by the prolonged and high-amplitude stimulus application needed to overcome behavioral habituation and sleep. The use of yoked test subjects that are stimulated randomly relative to sleep bouts is an important control for stress effects. However, data using this methodology have thus far only yielded modest effects of sleep deprivation on sleep rebound (Yokogawa et al., 2007).

Technical issues aside, an important, unresolved scientific issue is whether the amount of sleep rebound is proportionate to the amount of sleep deprivation in zebrafish. Additionally, better-refined definitions of zebrafish sleep and more sophisticated methods of monitoring and quantifying sleep states will provide new possibilities to study whether sleep deprivation affects sleep quality. For example, it would be interesting to test the hypothesis that sleep deprivation increases the depth in addition to the duration of sleep rebound in zebrafish.

### Zebrafish arousal states

Whereas sleep and waking are relatively easy to define with objective behavioral criteria, specific arousal states are more difficult to characterize. An animal's arousal state can be characterized by: (1) changes in frequency or intensity of voluntary locomotor activity and (2) altered responsiveness to sensory or emotional stimuli (Pfaff et al., 2008). In addition to these general characteristics, arousal can be characterized by the specific behavioral outputs that it motivates, such as reward-seeking and sexual or courtship behaviors.

Arousal-associated changes in locomotor activity can be triggered by intense stimuli. For example, in response to sudden changes in light intensity (e.g., light to darkness over 10 ms), larval zebrafish exhibit a biphasic response that begins with a transient, high-amplitude movement followed by a sustained, low-amplitude increase in locomotor activity that persists for at least several minutes (Prober et al., 2006; Emran et al., 2010). In addition to external stimuli, arousal states are also triggered by physiological drives such as hunger, sex, and pain. For example, adult zebrafish respond to caloric restriction with the same bi-phasic behavioral response resulting from mammalian hunger; starved fish are initially hyperactive, but become lethargic after prolonged caloric restriction (Novak et al., 2005). Arousal states can also be manifested as goal-seeking behaviors that change the structure of spontaneous locomotor activity. Food-seeking behavior is readily measured in larval zebrafish, which begin to hunt for food almost as soon as they can swim. This behavior can be quantitatively described by a temporal sequence that begins with ocular angle convergence followed by a series of orienting “J-turns” and forward swimming toward the target (Borla et al., 2002; Gahtan et al., 2005; McElligott and O'Malley, 2005; Bianco et al., 2011). These eye and tail movements are distinct from routine, spontaneous movements, enabling the objective identification of a food-seeking arousal state in both free-swimming and partially restrained preparations.

Zebrafish exhibiting heightened locomotor activity can also exhibit enhanced sensory responsiveness, consistent with the behavioral criteria for arousal. For example, zebrafish exposed to a sudden change in water flow rate become hyperactive and respond more quickly to a repeat application of the flow stimulus (Yokogawa et al., 2012). Zebrafish also exhibit similarly enhanced responses to a whole-field visual motion stimulus, which is thought to be a crucial sensory cue underlying the behavioral response to water flow. Notably, flow-induced arousal did not affect responses to electroshock and touch stimuli, which is suggestive of distinct arousal states. Although this study provides evidence of sensory modality-specific arousal states, the timecourses and behavioral readouts of the other stimuli were substantially different from the flow-related stimuli, leaving open the interesting question of what determines the specificity of an arousal state.

## Sleep/arousal neuromodulatory systems in zebrafish

The neuroanatomical and neurochemical systems that regulate sleep and arousal in mammals are largely conserved in zebrafish (Figure 1B). One notable difference is that zebrafish lack midbrain dopaminergic neurons analogous to the mammalian vPAG and VTA (Holzschuh et al., 2001; Kaslin and Panula, 2001; Rink and Wullimann, 2002; McLean and Fetcho, 2004), although the less-studied mammalian dopaminergic A11 group, which is noted for its roles in sensorimotor function and the human sleep disorder restless legs syndrome (Mignot et al., 2002), has a likely homolog in zebrafish ventral diencephalic dopamine clusters (Ryu et al., 2007; Tay et al., 2011). Also, zebrafish do not have a layered cortex, a principal target of mammalian ascending arousal systems, although homology between mammalian cortical areas and zones in the zebrafish dorsal telencephalon have been proposed based on common molecular developmental patterns (Wullimann and Mueller, 2004). Basal forebrain and brainstem cholinergic neurons have not been clearly described in zebrafish larvae.

Importantly, the neuropharmacology of mammalian behavior is well-conserved in zebrafish (Rihel and Schier, 2012), and drugs or bioactive agents that affect sleep and/or arousal in mammals produce comparable effects in zebrafish. Zebrafish exhibit a dose-dependent decrease in locomotor activity when treated with known hypnotics and sedatives, including melatonin, GABA receptor agonists (e.g., benzodiazepines, barbiturates, diazepam), histamine H1 receptor antagonists, and α2 adrenergic receptor agonists (Zhdanova et al., 2001; Ruuskanen et al., 2005; Renier et al., 2007; Sundvik et al., 2011), and modafinil, a wakefulness-promoting drug that is used to treat human sleep disorders, increases wakefulness in zebrafish (Sigurgeirsson et al., 2011). More recently, an unbiased screen of nearly 4000 small molecules corroborated the roles of arousal and sleep modulators, including noradrenaline, serotonin, dopamine, GABA, glutamate, histamine, adenosine, and melatonin, in regulating zebrafish sleep/wake behavior (Rihel et al., 2010). Studies examining more specialized aspects of arousal such as sensorimotor responses have also confirmed the role of key monoaminergic systems, including dopamine and serotonin, in regulating arousal states in zebrafish (Burgess and Granato, 2007; Mu et al., 2012; Yokogawa et al., 2012).

### Hypocretin

The Hcrt neuromodulatory system is the best-characterized regulator of sleep and arousal in zebrafish, and we focus on these studies to illustrate the current discoveries, concepts and issues that arise when studying neuromodulators of zebrafish sleep and arousal.

The zebrafish *hcrt* gene encodes two structurally-related peptides homologous to mammalian Hcrt1 and Hcrt2 (Kaslin et al., 2004; Faraco et al., 2006). Among vertebrates including zebrafish, there is particularly high sequence homology near the C-terminus of each Hcrt peptide, which is the critical region for biological activity and receptor selectivity (Asahi et al., 1999; Darker et al., 2001; Lang et al., 2004). The zebrafish genome contains a single *hcrt receptor* ortholog (*hcrtr2*; previously named *hcrtr*), a G-protein coupled receptor (GPCR) that is structurally similar to the two mammalian *hcrt receptor* paralogs (Prober et al., 2006; Yokogawa et al., 2007).

**Figure 3:**
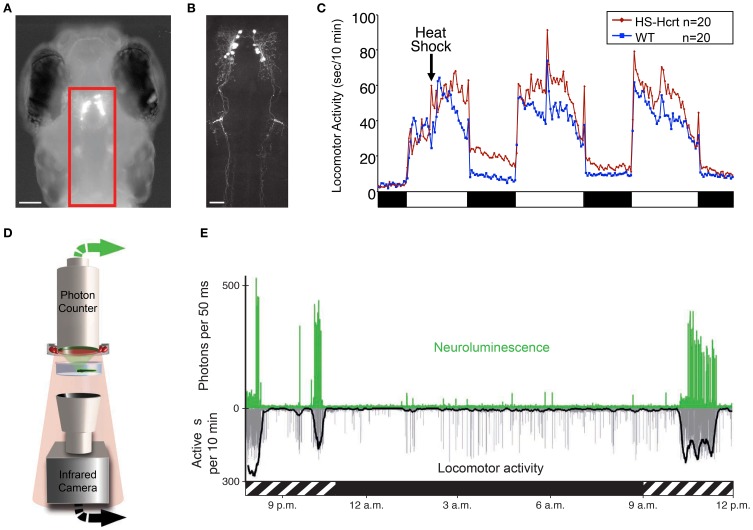
**Zebrafish hypocretin is associated with arousal. (A)** Dorsal view of a 4 dpf zebrafish larva that expresses GFP-Aequorin (GA) specifically in Hcrt neurons. **(B)** Two-photon z-projection image of the boxed area in **(A)**. Scale bars represent 100 μm **(A)** and 50 μm **(B)**. **(C)** Overexpression of Hcrt using a heat shock-inducible promoter (HS-Hcrt) increases locomotor activity. The mean locomotor activity of 20 HS-Hcrt larvae and 20 of their wild-type siblings is shown. The spike in activity during the afternoon of the 2nd and 3rd days of the experiment resulted from the addition of water to offset evaporation. **(D)** The GA assay. A large-area photon-counting photomultiplier tube is placed above a transparent behavior chamber in which a zebrafish larva that expresses GA in specific neurons is allowed to freely swim. The larva is imaged using infrared (IR) lights and an IR camera that is placed below the recording chamber. Spectral separation between GA neuroluminescence and the IR illumination allows the simultaneous recording of GA neuroluminescence and larval behavior. **(E)** Activity of Hcrt neurons during natural behavior. Data for a representative 4 dpf larva is shown. The larva exhibited periods of increased spontaneous locomotor activity (lower trace, thick line indicates 10 min running average) during the subjective day (hatched bar below graph) and little activity during the subjective night (black bar below graph). Most neuroluminescence signals produced by Hcrt neurons (upper trace) coincide with periods of robust locomotor activity during the subjective day, suggesting that Hcrt neuron activity is associated with arousal. Figure modified from Prober et al. (2006), Naumann et al. (2010).

At 5 dpf, when larval zebrafish sleep/wake behaviors are first observed, *hcrt* is specifically expressed in a bilateral nucleus in the posterior hypothalamus that encompasses ~10 neurons per hemisphere, as determined by *in situ* hybridization (ISH) and immunohistochemistry using a Hcrt1-specific antibody (Faraco et al., 2006; Prober et al., 2006). Furthermore, enhanced green fluorescent protein (EGFP) expression driven by various *hcrt* upstream promotor sequences faithfully recapitulates the endogenous *hcrt* expression pattern (Faraco et al., 2006; Prober et al., 2006) (Figure 3A). This cluster expands to approximately 40 neurons in the adult zebrafish hypothalamus (Kaslin et al., 2004; Appelbaum et al., 2009, 2010). While the zebrafish *hcrt* expression pattern is consistent with mammals, the number of *hcrt* neurons is on the order of 10^2^ fewer in zebrafish (de Lecea et al., 1998; Sakurai et al., 1998; Lin et al., 1999; Peyron et al., 2000; Prober et al., 2006). Two molecular markers of mammalian *hcrt* neurons, *vesicular glutamate transporter* and *neuronal pentraxin2*, also colocalize with *hcrt* in zebrafish (Appelbaum et al., 2009, 2010). Thus, the zebrafish provides a simple vertebrate system to study the development and function of Hcrt neurons.

Zebrafish *hcrt* neurons send widespread ascending and descending projections to brain areas associated with arousal (Figure 3B), as they do in mammals (Peyron et al., 1998; Taheri et al., 1999). Using a transgenic *hcrt*-EGFP line, Hcrt projections in larval zebrafish were found in close apposition to noradrenergic cells of the locus coeruleus and processes of diencephalic dopaminergic cells in larval zebrafish (Prober et al., 2006). By adulthood, Hcrt-immunoreactive fibers contact these targets and also densely innervate the serotonergic raphe and possibly histaminergic and cholinergic populations (Kaslin et al., 2004).

Consistent with widespread Hcrt neuron projections and with mammalian *hcrtr* expression, zebrafish *hcrtr2* expression is considerably more extensive than *hcrt* and is detected in widespread areas of the zebrafish brain. In larval zebrafish, one study used a high-resolution double fluorescent ISH method and found that *hcrtr2* colocalizes with *dopamine beta hydroxylase* in noradrenergic cells of the locus coeruleus and *dopamine transporter* in diencephalic dopaminergic cells (Prober et al., 2006), as in mammals (Trivedi et al., 1998; Marcus et al., 2001). However, another study concluded that there is no *hcrtr2* coexpression with these monoaminergic populations in 2 dpf or adult zebrafish, and instead reported that *hcrtr2* is expressed in GABAergic, adrenergic, cholinergic, and glycinergic systems (Yokogawa et al., 2007). The discrepancies reported in these studies may stem from differences in detection method, probe sensitivity and specificity, and possibly the developmental stages studied. For example, the latter study made extensive use of a two-color chromogenic ISH procedure, which cannot reliably report colocalization because the differently colored stains can mask each other, the colors cannot be spectrally separated into distinct channels, and high resolution imaging methods such as confocal microscopy cannot be used to resolve samples in three dimensions (Jowett and Yan, 1996; Vize et al., 2009; Lauter et al., 2011). Neither study observed expression of *hcrtr2* in histaminergic or serotonergic cells. However, the methods used in both studies, especially fluorescent ISH, often are not sensitive enough to detect low-abundance transcripts such as those encoding GPCRs, so negative data obtained using this method should be interpreted with caution. Indeed, a close examination of data obtained by the latter study suggests coexpression of faintly stained *hcrtr2* in some monoaminergic nuclei, consistent with the former study.

Several studies have also explored the functional role of the *hcrt* system in zebrafish. One study of larval zebrafish found that heat shock inducible overexpression of a *hcrt* transgene promotes wakefulness by consolidating active states, increasing arousal, and reducing sleep (Prober et al., 2006). Thus, the zebrafish Hcrt gain of-function (GOF) phenotype (Figure 3C) is comparable to the effects of intracerebroventricular injection of Hcrt peptide in rodents (España et al., 2001; Thakkar et al., 2001) and goldfish (Nakamachi et al., 2006). Conversely, sleep fragmentation is observed in adult zebrafish containing a null mutation in the *hcrtr2* gene (Yokogawa et al., 2007). This loss-of-function (LOF) result is strikingly similar to the sleep/wake fragmentation observed in rodents, canines and humans that lack Hcrt signaling (Sutcliffe and de Lecea, 2002). However, this study also made a controversial proposal that the zebrafish Hcrt system may be functionally divergent from the mammalian Hcrt system based on the observation that the *hcrtr2* mutant displayed a mild decrease in sleep, whereas Hcrt peptide injection caused a mild decrease in locomotor activity. However, the decreased sleep in *hcrtr2* mutants was only significant compared to unrelated, non-mutagenized animals, and the decreased locomotor activity following Hcrt peptide injection may have resulted from the relatively high doses of peptide used (280–2800 pmol/g body weight in adult zebrafish vs. 2.8–28 pmol/g body weight that increased locomotor activity in adult goldfish). Indeed, a subsequent study using the same *hcrtr2* mutant did not report a decreased sleep phenotype (Appelbaum et al., 2009). More recently, this debate seems to have been resolved by a report that inducible ablation of *hcrt* neurons using a genetically targeted toxin increased sleep and sleep/wake transitions in larval zebrafish (Elbaz et al., 2012). Thus, in non-invasive, inducible zebrafish systems, Hcrt GOF consolidates sleep/wake states and reduces sleep, whereas Hcrt LOF fragments sleep/wake states and increases sleep.

Taken together, the neuroanatomical and functional data from different research groups indicate that the zebrafish Hcrt system is interconnected with major neuromodulatory arousal systems and regulates sleep/wake transitions by promoting arousal. Thus, the zebrafish provides a simple model of the vertebrate Hcrt system. The zebrafish system is poised to tackle unanswered questions about how Hcrt regulates sleep and arousal states. For example, although the zebrafish data point to a role for Hcrt in arousal state regulation, its specific role in different forms of arousal is unknown. Zebrafish can also be used to characterize the neural mechanisms through which Hcrt affects sleep and arousal using GOF and LOF genetic tools such as heat shock inducible Hcrt transgenic zebrafish and the *hcrtr2* mutant in combination with mutant and transgenic zebrafish that lack other neuromodulatory systems. The relatively small number of Hcrt neurons should also facilitate studies of their development and connectivity, as well as analysis of their activity during different behaviors. Finally, small molecule screens in zebrafish can be used to identify therapeutic pathways for the treatment of narcolepsy.

## Up-and-coming approaches to studying sleep and arousal in zebrafish

Still in its infancy, zebrafish research on sleep and arousal has focused on establishing behavioral assays and identifying known regulators of behavioral states. Now that the zebrafish has been established as a useful model of vertebrate sleep and arousal, it is well-suited among commonly used model organisms to address two long-standing yet fundamental questions in sleep research. First, what are the genetic mechanisms that regulate sleep and arousal behaviors? Second, what are the neural mechanisms that underlie these behaviors?

### Discovery genetics and screens to identify sleep and arousal regulators

Progress in understanding mechanisms that regulate mammalian sleep has been limited by the challenge of performing unbiased screens in mammals. This limitation is underscored by recent progress in understanding genetic and neuronal mechanisms that regulate *Drosophila* sleep through the use of screens (Cirelli et al., 2005; Koh et al., 2008; Stavropoulos and Young, 2011; Pfeiffenberger and Allada, 2012; Rogulja and Young, 2012). In contrast to mammals, zebrafish are uniquely well-suited among vertebrate model systems for large-scale and unbiased screens. Indeed, two large-scale forward genetic screens for developmental and simple behavioral phenotypes stimulated the widespread adoption of zebrafish as a model system (Driever et al., 1996; Haffter et al., 1996). These screens used chemical mutagenesis to induce point mutations, and embryos and larvae were then screened for recessive phenotypes. The identification of a large number of developmental and behavioral mutants established zebrafish as the only vertebrate model system in which such screens are practical, and many labs have subsequently performed screens for a wide range of phenotypes.

However, there are several limitations in using forward genetics to identify genes that affect complex and quantitative behaviors such as sleep. First, as in mammals, sleep varies considerably among individual zebrafish, making it difficult to identify a population of individuals that exhibit a recessive phenotype. Second, mapping genes responsible for recessive phenotypes requires a significant amount of time and labor, and it can be challenging for variable and quantitative phenotypes. Recent advances in deep sequencing technologies can accelerate this process (Obholzer et al., 2012; Miller et al., 2013), but they do not reduce the challenge of mapping a variable quantitative trait such as sleep. These two factors likely underlie the relative paucity of genes that have been identified by *Drosophila* forward genetic screens despite large-scale efforts in several labs. Third, chemical mutagens typically induce thousands of mutations in each animal, and this high background mutational load may affect behavior. These problems can be avoided by performing insertional mutagenesis using retroviruses and transposons (Golling et al., 2002; Varshney et al., 2013), but these mutagens are much less efficient at inducing mutant phenotypes. This limitation is particularly problematic for screens that use relatively low-throughput assays, such as those required to identify sleep defects.

Two approaches have been developed that overcome some of the limitations of forward genetic screens. First, gene trap approaches use a modified transposon that creates a fluorescent fusion protein when it inserts within a gene (Clark et al., 2011; Maddison et al., 2011; Trinh et al., 2011). In some methods (Maddison et al., 2011; Trinh et al., 2011), the transposon can be induced to undergo genetic recombination, which creates a premature stop codon in the trapped gene. This approach is particularly useful when laborious screening assays are necessary because it allows for pre-selection of lines that are considered likely to be involved in the process of interest. For example, lines that exhibit fluorescence in specific neural populations, but not those that fluoresce in skin or muscle, can be selected for behavioral analysis. Because recombination within the transposon is inducible, the targeted gene can be mutated in specific cells and at specific times. This feature is useful for genes that have different functions in different cell types or are required for development. However, this technique often fails to create null mutants, and preselecting genes based on their expression pattern introduces a bias into the screen.

A second discovery approach that has recently been applied to zebrafish is the use of small molecule libraries to screen for behavioral phenotypes. Zebrafish larvae readily take up small molecules that are added to the water (Peterson and Fishman, 2004) and lack a mature blood brain barrier (Jeong et al., 2008). In addition, the small size of zebrafish larvae allows screening in 96 or 384 well-plates, and thousands of embryos can be routinely collected, which allows large numbers of compounds to be tested quickly. Taken together, zebrafish larvae allow physiologically relevant whole-animal assays combined with high-throughput, low-cost drug screening, making them perhaps the most suitable multicellular model organism for small molecule screens. This approach has led to the discovery of mechanisms that regulate development (North et al., 2007; Yu et al., 2008) and behavior (Kokel et al., 2010; Rihel et al., 2010; Wolman et al., 2011), and to the development of clinically relevant compounds (North et al., 2007). A significant challenge of small molecule screens is that it can be difficult to identify the targets of poorly annotated compounds. However, this problem will become less acute with the growth of databases that compile the effects of small molecules in a wide range of biological assays (Lamb et al., 2006; Tolopko et al., 2010) and the development of chemical bioinformatic approaches (Laggner et al., 2012).

As an alternative to forward genetic and chemical approaches, reverse genetic techniques have undergone rapid growth over the last few years and promise to transform the use of zebrafish in the genetic analysis of behavior. One method, known as TILLING (Kettleborough et al., 2011), uses chemical mutagenesis to create thousands of mutant zebrafish, each of which is then screened for mutations in a gene of interest using deep sequencing. This approach is expected to identify null mutations in most zebrafish genes within the next few years, although it will likely fail to identify mutations in many small genes. A second approach uses zinc finger nuclease (ZFN) and TAL-effector nuclease (TALEN) technologies (Bogdanove and Voytas, 2011). These nucleases can be designed to create double-stranded DNA breaks at specific sites in the genome, which are repaired by an error-prone process that often generates short insertions or deletions. TALENs are emerging as the superior technology because they are easy to generate, can target essentially any DNA sequence and are generally more mutagenic than ZFNs (Huang et al., 2011; Cade et al., 2012; Dahlem et al., 2012; Moore et al., 2012; Chen et al., 2013). Additionally, high-throughput methods (Briggs et al., 2012; Reyon et al., 2012; Wang et al., 2012) should allow the synthesis of TALEN libraries that target every zebrafish gene. Through a combination of these reverse genetic approaches, it is likely that a knock-out mutant will be available for essentially all zebrafish genes within the next few years. Large collections of these mutants can then be screened for behavioral phenotypes. Finally, TALENs can also be used to insert exogenous sequences into the zebrafish genome (Bedell et al., 2012). This technology should eventually allow the insertion of recombination sites, epitope tags and reporter genes at precise sites in the genome, which will enable sophisticated genetic approaches that are currently limited to more established genetic models such as mice, flies, and worms. A disadvantage of these approaches is that they preclude behavioral analysis of genes that are required for development. An alternative reverse genetic technique that can overcome this problem uses short hairpin RNAs (shRNAs) to target specific mRNAs for degradation. shRNA expression can be controlled in a spatial and temporal manner through the use of appropriate expression systems, thus avoiding complications for genes whose function is required during development or for different functions in different cell types. While this technology is not yet fully developed in zebrafish (De Rienzo et al., 2012), it is encouraging that it has been successfully used to identify genes that regulate sleep in flies (Rogulja and Young, 2012) and we expect that shRNA libraries that target all zebrafish genes will soon be developed.

### Characterization of neural circuit structure and function

The complexity of mammalian neural circuits and the difficulty of manipulating these circuits present major challenges in deciphering the neural mechanisms that regulate mammalian sleep and arousal. In contrast, the optical transparency and conserved yet relatively simple nervous system of zebrafish larvae present an opportunity to characterize the basic neural mechanisms that regulate vertebrate sleep. Zebrafish larvae are commonly used to monitor the development of particular neurons during development through the genetic targeting of fluorescent proteins. While useful, this approach is generally limited to visualizing individual neurons using transient plasmid injections, or all neurons of a genetically defined circuit using stable transgenic animals. However, it is difficult to simultaneously monitor the development of each neuron of a genetically specified neural population using a single fluorescent protein. A new transgenic tool, referred to as Brainbow, promises to overcome this limitation. Brainbow uses Cre/Lox recombination to generate the expression of random combinations of green, red and blue fluorescent proteins (Livet et al., 2007). The presence of multiple copies of the Brainbow transgene generates a large palette of colors that allows the axonal and dendritic projections of up to 100 neurons to be distinguished from their neighbors. While initially developed for the mouse, the poor accessibility of mouse brains for imaging limits the use of Brainbow in living mice, but zebrafish larvae are well-suited for this approach (Pan et al., 2011). The original Brainbow transgene is limited by use of the relatively dim cerulean fluorescent protein and the cytoplasmic localization of all three fluorescent proteins. However, we expect that use of membrane-targeted, brighter and more photostable fluorescent proteins will allow the axonal and dendritic projections of individual neurons among genetically specified neural populations to be characterized at the single cell level in developing zebrafish larvae.

A variety of transgenic tools have recently been developed that allow the stimulation or inhibition of genetically defined neural populations. So-called “optogenetic” tools allow genetically specified neurons to be stimulated or inhibited by specific wavelengths of light (Mattis et al., 2012). In principle, zebrafish larvae are well-suited for this technology due to their optical transparency, which is a significant advantage over non-transparent organisms such as rodents, which typically require the use of fiber optics to deliver light to specific brain regions. Indeed, this technology has been used to functionally characterize the roles of specific neurons in sensory and motor control in restrained zebrafish larvae (Douglass et al., 2008; Arrenberg et al., 2009; Wyart et al., 2009). However, zebrafish larvae exhibit robust behavioral responses to the light stimulus, which can be problematic for experiments using freely behaving larvae (Zhu et al., 2009). Alternative approaches using transgenes that modulate neural activity in the presence of specific small molecules (Szobota et al., 2007; Arenkiel et al., 2008; Alexander et al., 2009; Magnus et al., 2011) or at specific temperatures (Pulver et al., 2009) provide alternative approaches that avoid the confounding effects of light, but have yet to be tested in zebrafish.

### Neurophysiological correlates of sleep and arousal

Electrophysiology is often used to record neural activity during mammalian sleep/wake behaviors, but the available techniques are invasive and it is difficult to target specific neuron types for recording. While it is not feasible to perform electrophysiological neural recordings on freely behaving zebrafish larvae due to their small size, two alternative approaches may be used. First, fluorescent genetically encoded calcium indicators (GECIs) such as GCaMP report cellular calcium levels, which can be used as a proxy for neural activity (Akerboom et al., 2012). An important advantage of using GECIs is that cell-type specific promoters can be used to target their expression to specific neuron types for recording. Alternatively, GECIs can be broadly expressed in many neurons, and the activities of individual neurons can be spatially resolved in the context of anatomical or molecular markers. Because zebrafish larvae are transparent, changes in GECI fluorescence can be monitored in essentially any neuron in intact larvae, and many neurons can be monitored simultaneously (Ahrens et al., 2012). A disadvantage of this technique is that animals must be restrained to maintain a stable image of neural activity, although a closed-loop virtual reality system recently developed for head-restrained larvae augments the behavioral repertoire that can be recorded simultaneously with neural activity (Portugues and Engert, 2011; Ahrens et al., 2012). However, using GECIs to monitor neural activity during sleep/wake behaviors is particularly problematic because the light that is required to excite GECI fluorescence will affect behavior.

An alternative approach that was recently developed for zebrafish larvae and avoids some limitations of GECIs uses a GFP-Aequorin (GA) reporter (Naumann et al., 2010). This technique is non-invasive, does not require excitation light and allows an animal to freely behave while the activities of genetically specified neurons are monitored. In the presence of a cofactor and the increased levels of cytoplasmic calcium that accompany neural activity, neuronally-expressed, luminescent GA emits photons (neuroluminescence) that can be detected from a recording chamber by a large-area photon counter (Figure 3D). Simultaneously, behavior is monitored using an infrared camera. The utility of this technique was validated using transgenic zebrafish expressing GA specifically in Hcrt neurons (Figures 3A,B). Strikingly, Hcrt neuron neuroluminescence corresponds to peak periods of locomotor activity that occur during the subjective daytime (Figure 3E), suggesting that Hcrt neuron activity is correlated with arousal. These results are similar to the limited *in vivo* data obtained from identified Hcrt neurons in restrained and behaving rats (Lee et al., 2005; Mileykovskiy et al., 2005). A limitation to the non-imaging GA approach is that it does not provide spatial information about the neurons that are producing bioluminescent signals. It is therefore necessary to restrict GA expression to the neurons of interest, which is currently only possible for a small number of neural populations where a cell type-specific promotor has been identified. Particularly troublesome is the observation that promoters used to drive gene expression in neurons often also drive gene expression in muscle, although inclusion of a neuron-restrictive silencing element (NRSE) adjacent to the promoter sequence can significantly reduce non-neural gene expression (Bergeron et al., 2012). Another limitation to this technique is that it is limited to monitoring individual larvae, although it may be possible to scale up the assay using multiwell plates and PMT arrays or an electron-multiplying CCD camera. Thus, while this technique is still in development, it has the potential to transform studies of neural circuit activity, function and behavior in transparent and genetically tractable model organisms such as zebrafish.

## Concluding remarks

These are still early days for using zebrafish as a model to study sleep and arousal states, but it is already clear that zebrafish can provide new and important insights into genetic and neural mechanisms that regulate behavior. High-throughput screens have revealed new mechanisms that regulate arousal (Kokel et al., 2010; Rihel et al., 2010), and tools that exploit advantageous features of zebrafish have provided new insights into the neural regulation of sleep and arousal (Naumann et al., 2010; Yokogawa et al., 2012). The development of new tools to monitor and manipulate neural activity, as well as technologies that allow precise genome editing, are rapidly increasing the scope of experiments that can be performed with zebrafish. We expect that these developments will lead to the discovery of new mechanisms that regulate sleep and arousal states that would be difficult to obtain using other vertebrate model organisms. Finally, the utility of zebrafish for performing high-throughput whole animal small molecule screens may lead to new therapies for sleep and arousal disorders.

### Conflict of interest statement

The authors declare that the research was conducted in the absence of any commercial or financial relationships that could be construed as a potential conflict of interest.
